# Fostering empathy in healthcare: impact of a simulation-based educational program on professionals’ relational skills

**DOI:** 10.3389/fmed.2026.1881591

**Published:** 2026-08-25

**Authors:** Lidia Borghi, Maria Adelaide Morabito, Elaine C. Meyer, Philip Scilio, Elena Vegni, Giulia Lamiani

**Affiliations:** 1 Department of Health Sciences, University of Milan, Milan, Italy; 2 Unit of Clinical Psychology, Santi Paolo and Carlo Hospital, Milan, Italy; 3 Department of Psychology, Catholic University of the Sacred Heart, Milan, Italy; 4 Center for Bioethics, Harvard Medical School, Boston Children’s Hospital, Boston, MA, United States

**Keywords:** empathic attitude, healthcare professionals, Jefferson scale, continuous professional development, medical education

## Abstract

**Introduction:**

Empathy has been recognized as a crucial quality for patient-centered care and is associated with improved therapeutic outcomes and greater satisfaction of patients and clinicians. However, empathy may decline over the course of clinical practice, highlighting the need for effective educational interventions.

**Methods:**

The study examined changes in empathic attitude among healthcare professionals participating in the Italian simulation-based Program to Enhance Relational and Communication Skills (PERCS-Italy) across three hospitals in Northern Italy between 2019 and 2024. The Jefferson Scale of Empathy (JSE) was administered to participants before and after attending PERCS-Italy workshops.

**Results:**

A total of 303 healthcare professionals completed the pre-post training JSE assessments. Empathic attitudes increased significantly after the workshops (*t* = −4.72, *p* < 0.001). The observed increase in empathic attitude following participation in PERCS-Italy was similar across gender and clinical discipline. Additionally, an inverse correlation between years of professional experience and empathic attitude was observed.

**Discussion:**

The study reinforces the potential value of the PERCS-Italy program in increasing healthcare professionals’ relational qualities, particularly empathy.

## Introduction

Empathy is a crucial human quality for patient-centered care, as it allows healthcare professionals to understand patients’ perspectives, including needs, values, and preferences, thereby strengthening trust and therapeutic alliance (1). Empathy was first defined by Rogers in 1959 (2) as the state “to perceive the internal frame of reference of another with accuracy, and with the emotional components and meanings which pertain thereto, as if one were the other person, but without ever losing the ‘as if’ condition” (p. 210). In healthcare settings, recent literature has emphasized the construct of clinical empathy (3, 4), that involves an understanding of the patient’s feelings and perspectives, combined with the capacity to communicate this understanding and the intention to help. Within this framework, empathy is not merely an individual disposition, but it is believed to be a professional competence (4), related to positive effects on patient satisfaction and health outcomes, such as patient self-efficacy, treatment adherence, and disease management planning (5), as well as the emotional wellness of healthcare workers (3, 6). Interestingly, clinicians’ empathy has been associated with occupational well-being, such as higher job satisfaction (7), while systematic review evidence suggests a negative association between burnout and empathy in healthcare professionals (8). For this reason, empathy is also considered a fundamental quality of clinicians in the context of well-developed communication and relational skills to provide patient-centered care (9, 10).

However, empathy is vulnerable to erosion during health professions education and clinical practice, particularly in high-pressure contexts, making its development and maintenance an educational and organizational priority (11).

In recent years, several educational programs have been developed to enhance healthcare professionals’ and students’ communication and relational skills (12–16). Among these interventions, the Program to Enhance Relational and Communication Skills (PERCS) emerged as a unique interdisciplinary educational model to cultivate clinicians’ communication skills and relational capacities, while increasing perceived preparedness and confidence in complex clinical conversations with patients and families (17, 18). The rationale of PERCS is grounded in an integrated theoretical framework that combines relational learning, experiential learning, reflective practice, and simulation-based medical education. The PERCS model was originally developed from the perspective of relational learning (19), which is based on the premise that professional development in healthcare professionals occurs within the context of relationships established among practitioners, patients, and family members. PERCS was also designed to address the *hidden curriculum*, namely the discrepancy between what is taught in formal educational settings and what practitioners actually learn through the informal flow of professional training and everyday clinical practice (20–22). Accordingly, this approach views professional competence as embedded in the moral and emotional dimensions of clinical practice (23), shifting the focus of learning from content alone to process and relationships (24). Consistent with experiential learning theory (25), participants engage in realistic simulated encounters followed by feedback, facilitated reflection, and debriefing, allowing them to transform experience into learning and to experiment with new ways of responding in practice (26–28). Through these experiential learning methods, the PERCS model aims to cultivate reflective and relational capacities such as self-awareness, confidence and self-efficacy, tolerance of uncertainty, and a sense of empathy toward patients and families (22). The rationale of PERCS is supported by evidence from other educational programs. Regarding its educational underpinnings, experiential, role-play–based communication skills training has been shown to improve clinicians’ empathic skills (29–31). Evidence also supports the relevance of the individual capacities targeted by PERCS. Self-awareness is conceptually regarded as foundational to the therapeutic relationship (32) and has been associated with greater empathy in healthcare professionals (33). Similarly, greater self-efficacy has been qualitatively linked to “empathic bravery” defined as the willingness and eagerness to engage empathically with patients (34). Furthermore, among medical trainees, changes in tolerance of ambiguity have been positively associated with changes in empathy (35).

However, limited empirical evidence has examined whether participation in PERCS-Italy is associated with changes in healthcare professionals’ empathy.

The study aimed to evaluate whether participation in the PERCS-Italy program was associated with changes in healthcare professionals’ empathic attitudes. Since the empathy of healthcare professionals decreases over time (11, 37), and female healthcare professionals have been found to have higher empathy scores than male healthcare professionals (38–40), we expected that changes in empathic attitude following participation in the PERCS-Italy program might vary by years of professional experience and gender. We also examined whether these changes varied across clinical disciplines.

## Materials and methods

### The PERCS-Italy program

The PERCS program was originally developed in 2002 at Boston Children’s Hospital (22). In 2008, following a period of collaboration between Boston Children’s Hospital and University of Milan, the PERCS program was adapted to the Italian context (PERCS-Italy) (36). The PERCS-Italy program has been offered as a continuing medical education program at Santi Paolo and Carlo Hospital and, later, at Humanitas Research Hospital (18). Over the time, various PERCS-Italy workshops customized to different clinical settings and communicative demands have been developed, such as, among others, PERCS-Emergency Medicine, PERCS-Medical Error, PERCS-Organ Donation, PERCS-Oncology, and PERCS-End of Life (18).

PERCS-Italy workshops, regardless of topic and clinical context, have been implemented with the same educational pedagogy, experiential learning principles, and structure. Each workshop lasts approximately 5 h and enrolls a maximum of 10–15 interdisciplinary professionals. The workshops are generally conducted by two facilitators with training in clinical psychology and experience in healthcare communication. The format of PERCS workshops is consistent and includes the following phases: (1) warm-up phase, where principles of PERCS are presented and participants introduce themselves; (2) brainstorming, aimed at sharing pre-existing knowledge and suggestions about the elements that enable good communication in the participants’ setting; (3) didactic lecture focused on communication and relational skills for the particular PERCS topic or setting; (4) simulations and group debriefings aimed to foster self-reflection and active experiential learning; and (5) cognitive integration phase, with participant-generated take-home points and reflections to consolidate the learning.

The core educational elements of PERCS workshops include enactments of case scenarios with patients and family members portrayed by actors and voluntary clinician-participants, and subsequent debriefings. The case scenarios are developed with clinical experts to ensure a realistic representation of difficult situations for the topic of the workshop. An example of a case scenario used in PERCS-Oncology is provided in the Appendix, Table A1. In the simulations, one or two volunteers participate in their familiar clinical roles. Volunteers are asked to interact naturally as they typically would in their actual professional roles throughout the simulation to enhance realistic, relevant learning. Participants who are not directly involved in the simulations learn by observing and exchanging their perspectives. Indeed, in PERCS training, collaborative learning arises not only from the interdisciplinary composition of the group, but also from the different positions participants occupy during the simulation, which bring complementary aspects of the same encounter into view (22). Consistently, learners in observer roles have been found to achieve outcomes comparable to those of active participants in communication-focused simulations (41), particularly when observers are actively engaged and contribute to the debriefing (42). There are two simulations in total, and debriefings are conducted at the conclusion of each one, involving all participants and facilitators. Details on how debriefings are conducted are available elsewhere (43). PERCS-Italy workshops conclude with the sharing of take-home messages by each participant to summarize and consolidate the learning experience (18).

Before and immediately after the workshops, self-report questionnaires were administered to participants to assess learning. Since 2019, the evaluation of the potential value of PERCS-Italy program was conducted by administering the Jefferson Scale of Empathy (JSE) before and after the workshops. The Jefferson Scale of Empathy was selected because it is a content-specific and context-relevant instrument developed to assess empathy in health professions (40, 44, 45).

### Data collection

The recruitment and enrollment to PERCS-Italy workshops were accomplished through the hospitals’ intranet. The enrollment was on a voluntary basis. The PERCS-Italy workshops were open to all interested professionals (e.g., physicians, nurses, psychosocial professionals, administrators) of the hospitals involved, without any exclusion criteria.

At the beginning of each workshop, participants’ socio-demographic data (e.g., gender, age, nationality, professional role, and years of experience) were collected.

To assess participants’ empathic attitude, the Italian paper-based version (46) of the Jefferson Scale of Empathy – JSE (version for physicians and other healthcare professionals) was administered immediately before and after the workshops. The JSE consists of 20 items; each measured on a seven-point Likert-type scale from 1 (strongly disagree) to 7 (strongly agree). Half of the items are directly scored (e.g., “My patients feel better when I understand their feelings”); half are reverse scored (e.g., “I believe that emotion has no place in the treatment of medical illness”). The total score ranges from 20 to 140, with higher scores indicating greater empathic attitude.

To reduce social desirability bias, participants completed the questionnaires anonymously and were informed that responses would be used exclusively for educational evaluation and research purposes in aggregated form. Questionnaire completion was voluntary, and no identifying information was linked to individual responses.

The JSE was used in this study by permission from Thomas Jefferson University, which holds the copyright of the scale.

### Data analysis

Participants’ socio-demographic characteristics and their distribution across different PERCS-Italy workshops were analyzed through descriptive statistics. *T*-tests for paired samples were conducted to examine the difference in participants’ scores on JSE before and after the workshops. To measure changes in empathic attitude following participation in PERCS-Italy workshops, the difference between post-pre JSE total scores (∆ = posttest – pretest) was calculated. Independent *t*-test analysis was performed on JSE ∆ score in gender groups (male vs. female). Pearson correlation was conducted to explore the correlation between the JSE ∆ score and the years of experience. A one-way ANOVA was performed on JSE ∆ scores across professional groups (physicians vs. nurses vs. psychosocial professionals vs. other professionals).

## Results

### Participants

A total of 400 interdisciplinary participants enrolled in the PERCS-Italy workshops, of which 303 fully completed the pre-post JSE questionnaires, yielding a 76% (303/400) data completion rate.

Between 2019 and 2024, 42 PERCS-Italy workshops were delivered across the three participating hospitals (San Paolo and Carlo Hospital *n* = 10; Humanitas Research Hospital *n* = 30; Policlinico Hospital *n* = 2), spanning 10 thematic areas: PERCS-Dialysis (*n* = 1), PERCS-Oncology (*n* = 8), PERCS- Medical Error (*n* = 12), PERCS- End of Life (*n* = 6), PERCS-Covid (*n* = 2), PERCS-Organ Donation (*n* = 4), PERCS-Aggression (*n* = 4), PERCS-Neurosurgery (*n* = 2), PERCS-Intensive Care Unit (PERCS-ICU) (*n* = 1), and PERCS-Radiology (*n* = 2). Socio-demographic characteristics of all PERCS-Italy participants (both completers and non-completers) along with the distribution of participants across the PERCS workshop types are reported in Table 1. Completers and non-completers did not differ significantly in years of experience, age, nationality, or hospital, whereas significant differences emerged for professional discipline (χ^2^ = 27.1, *p* = 0.001) and gender (χ^2^ = 4.3, *p* = 0.04). Detailed comparisons are also reported in Table 1.

**Table 1:** Participants’ socio-demographic characteristics and distribution across PERCS-Italy editions.

Participants’ characteristics	Total sample (*n* = 400)	Valid sample for analysis (*n* = 303)	Sample with missing data (*n* = 97)	Statistics
Discipline, *N* (%)				*X*^2^ = 27.1; *p* = 0.001
Physicians	180 (45.4)	146 (48.5)	34 (35.8)	
Nurses^a^	139 (35.1)	107 (35.5)	32 (33.7)	
Psychosocial professionals^b^	14 (3.5)	12 (4)	2 (2.1)	
Other professionals^c^	63 (16)	36 (12)	27 (28.4)	
Valid N	396 (100)	301 (100)	95 (100)	
Years of experience, mean (sd), range	13.6 (12.0), 0–43	13.8 (11.8), 0–43	13.3 (13.1), 0–39	*t* = −0.27, *p* = 0.79
Valid N	324	255	69	
Age, mean (sd), range	40.01 (12.1), 22–68	40.1 (11.8), 22–68	39.7 (13.0), 23–65	*t* = −0.31, *p* = 0.76
Valid N	389	296	93	
Gender, *N* (%)				*X*^2^ = 4.3; *p* = 0.04
Male	121 (30.6)	100 (33.3)	21 (22.1)	
Female	274 (69.4)	200 (66.7)	74 (77.9)	
Valid N	395 (100)	300 (100)	95 (100)	
Nationality, *N* (%)				*X*^2^ = 3.2; *p* = 0.199
*Italian*	320 (93.9)	247 (95)	73 (90.1)	
European	10 (2.9)	7 (2.7)	3 (3.7)	
Extra-European	11 (3.2)	6 (2.3)	5 (6.2)	
Valid N	341 (100)	260 (100)	81 (100)	
Hospital, *N* (%)				*X*^2^ = 5.7; *p* = 0.12
San Paolo and Carlo	88 (22)	66 (21.8)	22 (22.7)	
Humanitas	282 (70.5)	209 (69)	73 (75.2)	
Policlinico	30 (7.5)	28 (9.2)	2 (2.1)	
Valid N	400 (100)	303 (100)	97 (100)	
Types of PERCS, *N* (%)				
PERCS-Dialysis	6 (1.5)	6 (2)	0 (0)	
PERCS-Oncology	68 (17)	47 (15.5)	21 (21.6)	
PERCS-Medical Error	100 (25)	72 (23.8)	28 (28.9)	
PERCS-End of Life	52 (13)	34 (11.2)	18 (18.6)	
PERCS-Covid	15 (3.8)	11 (3.6)	4 (4.1)	
PERCS-Organ Donation	40 (10)	37 (12.2)	3 (3.1)	
PERCS-Aggression	53 (13.3)	40 (13.2)	13 (13.4)	
PERCS-Neurosurgery	26 (6.5)	20 (6.6)	6 (6.2)	
PERCS-ICU	11 (2.7)	11 (3.6)	0 (0)	
PERCS-Radiology	29 (7.2)	25 (8.3)	4 (4.1)	
Valid N	400 (100)	303 (100)	97 (100)	

^a^Includes nurses and midwives; ^b^This category includes psychologists, educators, social workers, and chaplains; ^c^This category includes, lab technicians, pharmacists, risk managers, and administrators.

### Empathic attitude

After PERCS-Italy workshops, participants self-reported a statistically significant increase in empathic attitude, which represents a score increase of 3 points (*t* = −4.72, *p* < 0.001), with a small effect size (Cohen’s *d* = −0.27; CI: −0.39−−0.16), as reported in Table 2.

**Table 2:** Healthcare professionals’ JSE scores before and after the PERCS-Italy program.

Measure of empathic attitude	Pre-test Mean (sd)	Post-test Mean (sd)	Paired-sample *t*-test	*p*-value	Cohen *d*	95% confidence interval
JSE score	109.7 (12.29)	112.7 (12.38)	−4.72	<0.001	−0.27	−0.39 − −0.16

**Figure 1:**
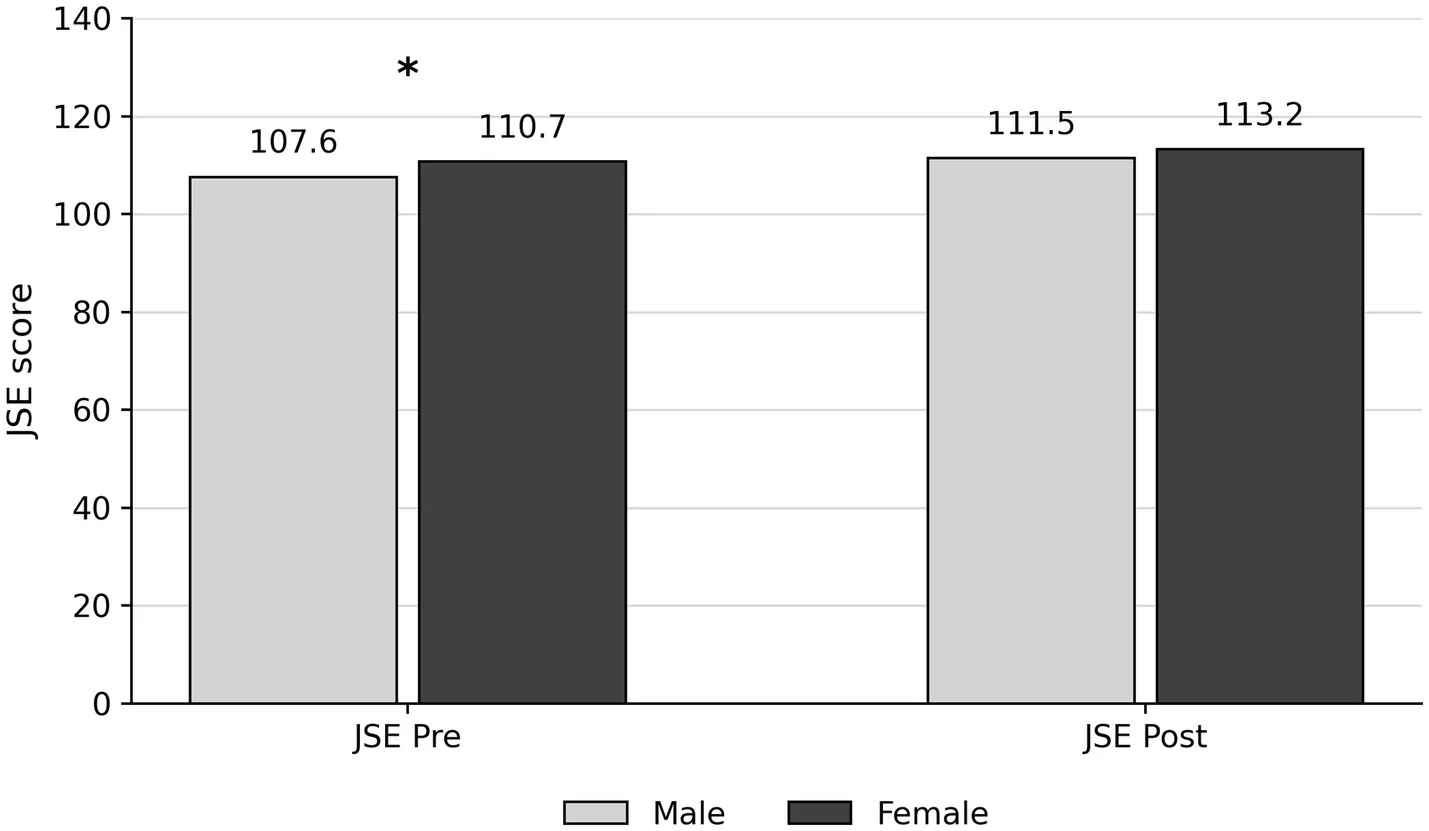
Pre- and post-training JSE scores by gender. *p < 0.05 for the difference between male and female participants at pre-test.

Regarding the analyses across gender, male participants showed significantly lower (*t* = −2, *p* = 0.022) JSE scores at pre-test (mean = 107.6) compared to female participants (mean = 110.7). However, after participation in PERCS-Italy, JSE scores increased significantly for both male (mean = 111.5) and female participants (mean = 113.2), with no gender difference at post-test (*t* = −1.2, *p* = 0.122) (Figure 1).

Accordingly, *t*-tests on JSE ∆ scores across gender showed no significant effect, as reported in Table 3 (*t* = 0.92, *p* = 0.178).

**Table 3:** JSE ∆ scores across gender and discipline group.

Variable	Mean (sd)	Test	*p*-value	Cohen d	95% confidence interval
Gender		*t* = 0.92	0.178	0.11	−0.13 − 0.35
Male	3.8 (11.8)				
Female	2.6 (10.8)				
Discipline group		*F* = 1.11	0.343	0.01	0.00–0.37
Physicians	4.1 (12.3)				
Nurses	1.5 (10.2)				
Psychosocial professionals	3.1 (6.5)				
Other professionals	3.0 (9.8)				

Pearson’s correlation showed a negative association between the JSE ∆ score and the years of experience (*r* = −0.128, *p* = 0.041), indicating greater increases in JSE scores among participants with fewer years of professional experience. Pre-JSE scores were not found to correlate with years of experience (*r* = 0.034, *p* = 0.553).

Regarding disciplinary groups, pre- JSE scores were not found to differ across disciplines (physicians mean = 108.6 vs. nurses mean = 110.3 vs. psychosocial workers mean = 114.3 vs. other professionals mean = 110.6; *F* = 1.13, *p* = 0.337). The one-way ANOVA performed on JSE ∆ score across discipline groups showed that changes in empathic attitude following participation in the PERCS-Italy did not vary across participants’ discipline (*F* = 1.11, *p* = 0.343). Table 3 summarizes the analyses of JSE ∆ scores.

## Discussion

This study aimed to evaluate whether participation in the PERCS-Italy program was associated with changes in healthcare professionals’ empathic attitude, considering possible differences based on gender, years of clinical experience, and professional discipline.

The primary finding indicates that participation in PERCS-Italy was associated with a statistically significant increase in healthcare professionals’ empathic attitude post-workshop. Although the magnitude of change was small, this finding is educationally relevant given the type and sustainability of the intervention, such as the brief duration and the real-world implementation. This aligns with prior research suggesting that targeted educational programs can positively influence empathy levels (12, 15, 29–31) and other communication skills among healthcare professionals and students (14, 16). The pedagogical principles of PERCS, which integrate relational learning, experiential learning, reflective practice, and simulation-based medical education (19, 22) may have contributed to the observed changes, as suggested by previous studies emphasizing the importance of active engagement in empathy training (47–49). Moreover, some of the reflective and relational capacities targeted by PERCS, including self-awareness, confidence and self-efficacy, and tolerance of ambiguity, have also been associated with empathy in the literature (33–35). These findings further support the rationale for considering empathy as a relevant outcome of participation in the PERCS-Italy program. Interestingly, although the overall increase in healthcare professionals’ empathic attitude following participation in the PERCS-Italy was significant, subgroup analyses revealed no significant differences based on gender or professional discipline, and only a small but significant negative association between empathic attitude scores and years of experience.

Regarding gender, female participants showed significantly higher baseline empathic attitude scores compared to male participants, which is consistent with previous studies reporting gender differences in self-reported empathy (38, 39, 50). However, post-training scores showed no significant difference between genders, and the increase in empathic attitude (∆ JSE) did not differ significantly either. This suggests that changes observed after PERCS-Italy were comparable for both male and female participants, likely due to its inclusive and experiential format, which emphasizes universal humanistic competencies, relational awareness, and interpersonal communication skills. Our findings are consistent with recent evidence supporting the idea that experiential learning, such as role-playing simulations and narrative reflection, may foster empathy across diverse learner profiles, regardless of gender (31).

We also found a modest but statistically significant inverse correlation between years of experience and increase in empathic attitude. That is, less experienced healthcare professionals appeared to report a greater increase in empathic attitude than more experienced professionals, as previous research has shown (11, 37). This result is also consistent with evidence that emotional skills may vary by years of experience. Miguez-Torres et al. found that younger nurses showed a better ability to feel, express, and understand emotional states than the older ones; however, reported differences are generally small (51, 52). Yet, it is important to note that baseline JSE scores were not associated with years of experience, suggesting that while initial empathic attitude levels are comparable across seniority, the observed capacity for change may diminish over time, possibly due to entrenched communication habits or cynicism acquired through years of practice (53, 54).

Regarding changes in empathic attitude across disciplines, our findings indicate that the increase observed after PERCS-Italy was comparable across physicians, nurses, psychosocial workers, and other healthcare professionals. This aligns with literature highlighting empathy as a core competence across helping professions, with particular relevance to health and social care (55). Moreover, recent studies emphasize the value of interdisciplinary learning to support healthcare professionals’ communication skills and confidence, especially in dealing with difficult conversations with patients and families (56, 57). The uniform increase across disciplines observed in our study may reflect the interprofessional design of the PERCS workshops, which facilitate shared understanding, narrative exchange, and team-based debriefing, fostering empathy not as a specific personal trait but as a professional competence in healthcare. The development of empathic attitude should not only be an underlying objective in health and social care educational programs, but also a focus of lifelong and continuous professional education (55).

### Limitations and future directions

The study has some limitations that need to be discussed. First, the pre-post JSE completion rate was 76%, indicating that 24% of participants did not complete both questionnaires. To assess potential attrition bias, we compared completers and non-completers on baseline characteristics. The two groups did not differ significantly in years of experience, age, nationality, or hospital, whereas significant differences emerged for professional discipline and gender. This indicates that non-completion was not completely random, which may have introduced attrition bias and, consequently, a degree of selection bias in the analysed sample. Reasons for non-completion were not systematically recorded, which represents a further limitation. The incomplete follow-up may also be partly related to the COVID and post-COVID data collection period, during which several workshops were evaluated online and post-workshop questionnaires were more easily missed. Future studies should monitor reasons for non-completion and adopt strategies to improve follow-up response rates. Second, the primary analysis relied on a single, paired-samples comparison and is therefore not affected by multiple testing inflation. However, the subgroup analyses (gender, years of experience, and discipline) were exploratory and were not corrected for multiple comparisons; therefore, the corresponding findings should be interpreted with caution. Third, although the PERCS-Italy editions addressed different clinical topics, participants were pooled for the main analysis, as the smaller subgroups did not allow for reliable type-specific analyses. Subgroup sizes varied considerably; thus, pooling different PERCS workshop types may have introduced between-group variability related to specific clinical contexts and participant characteristics. Fourth, the role participants occupied during the simulations was not among the variables recorded; changes in JSE scores could therefore not be attributed to direct involvement in the enactment versus observation. Previous studies suggest that observers achieve outcomes comparable to those of active participants on other educational outcomes (41, 42); whether this also applies to empathic attitude remains to be established. Future studies comparing pre-post JSE scores between participants who enact the case scenario and those who observe would help clarify this point and further substantiate the collaborative learning underpinning the PERCS model (22). Furthermore, healthcare professionals’ empathic attitudes were assessed using a self-report instrument (JSE); therefore, findings should be interpreted as changes in self-perceived empathy (40, 44, 45) rather than as direct evidence of behavioral improvements in clinical practice or patients’ perceptions of healthcare professionals’ empathy. Although questionnaires were completed anonymously to reduce social desirability bias, participants may still have reported increased empathy due to greater awareness of expected responses after the workshop. Moreover, response shift bias cannot be excluded, as participants’ understanding of empathy may have changed after the educational experience. Future research should consider observational measures and/or patient feedback to complement self-assessment and provide a more comprehensive evaluation of how the intervention may affect clinicians’ behavior and patients’ perceptions of empathy (12–16). Additionally, the absence of a control group limits the possibility of attributing the observed changes in empathic attitude exclusively to the PERCS-Italy intervention. Alternative explanations, including, demand characteristics, and response shift bias, cannot be excluded. Future controlled studies are needed to determine the effectiveness of the intervention and to strengthen causal inference. Moreover, the sustainability of changes in empathy over time remains unknown. Longitudinal research should assess whether PERCS-related changes are maintained over time and whether they require reinforcement through continuous professional development, institutional support, ongoing mentorship, structural reinforcement, and positive ethical climates.

Finally, the educational program proposed here focused on individual healthcare professionals; however, previous research has shown a lack of organizational-level interventions for systematic changes of empathy (58). Further studies on institutional and organizational factors that facilitate or hinder the adoption and flourishing of empathy-enhancing interventions could contribute to the development of systemic strategies for promoting empathic healthcare practices.

### Practice implications

The findings of this study have important implications for both healthcare education and clinical practice. The study provides evidence that participation in the PERCS-Italy program was associated with an immediate increase in healthcare professionals’ empathic attitude. Although the effect was small, the findings are relevant because they support the value of integrating experiential, simulation-based, and reflective learning approaches into healthcare curricula and continuous professional development, in order to help mitigate the concerning decline in empathy often observed in clinical practice (11, 37). Moreover, enhancing empathy among healthcare professionals is crucial for patient-centered care, and it is associated with improved patient satisfaction, better adherence to treatment, and improved patient outcomes (59, 60).

### Innovation

This study contributes to the literature by providing empirical evidence that participation in the PERCS-Italy program was associated with an immediate increase in healthcare professionals’ empathic attitude. This finding is relevant because empathy has remained mostly underexplored as a specific outcome of PERCS-based training, despite being central to patient-centered care.

By leveraging a structured, simulation-based educational model grounded in realistic case scenarios and collaborative debriefing, participation in PERCS demonstrates that empathy can be actively cultivated through experiential and reflective learning processes. Importantly, the observed increase in empathy did not differ across gender and professional disciplines, suggesting that PERCS may be suitable for heterogeneous interprofessional groups in healthcare settings. Furthermore, the findings support a shift from viewing empathy as a static personal trait to conceptualizing it as a dynamic, trainable competency that can be strengthened throughout the professional lifespan. The integration of PERCS into both undergraduate medical education and continuing professional development represents a sustainable innovation to counteract the well-documented decline of empathy in clinical practice. Embedding such training within healthcare systems may not only enhance clinicians’ relational skills but also promote a more consistent adoption of patient-centered care, ultimately improving both patient outcomes and professional well-being.

## Data Availability

The raw data supporting the conclusions of this article will be made available by the authors, without undue reservation.

## Appendix

Table A1: Example of a case scenario used in PERCS-Oncology.

**Table:**

Case scenario – Monica Mantovani
Monica is a 45-year-old woman working as an office employee at a company that manufactures air-conditioning systems. She is separated and has two children, Veronica (16 years) and Giorgio (11 years). One year ago, during the preoperative work-up for elective inguinal hernia repair, a chest X-ray showed an abnormal finding. Further investigations led to a diagnosis of non-small cell adenocarcinoma of the left lung. After staging with total-body PET/CT, Monica underwent left pneumonectomy. The tumour was completely resected, and histological examination showed lymph node involvement (pT2 pN2 M0). Over the following year, she received adjuvant chemotherapy with cisplatin (CDDP) and vinorelbine (VNB); adjuvant radiotherapy was also proposed. Roberta, Monica’s sister, sought information about the disease from the outset and has accompanied Monica throughout treatment and at every follow-up visit.

**Table:**

First conversation: six months after the end of treatment	Second conversation: six months after the start of the new treatment
Monica has undergone a follow-up CT scan, which shows mediastinal and supraclavicular lymphadenopathy. Monica will start first-line chemotherapy for recurrent advanced disease. Docetaxel (Taxotere) will be administered weekly for three weeks out of four, with corticosteroid premedication to prevent hypersensitivity reactions (taken at home the evening before and on the morning of each administration). If treatment is well tolerated, disease will be reassessed after three cycles. This regimen may cause hair loss and, unlike the previous one, may cause allergic reactions and hematological toxicity. The physician and the nurse meet Monica and Roberta to communicate the results of the follow-up visit and the proposed subsequent treatment.	Docetaxel chemotherapy was well tolerated and was followed by targeted therapy with erlotinib (Tarceva). However, reassessment shows that the disease is progressing. One week ago, Monica was taken to the Emergency Room following a seizure. Brain metastases are multiple, and one of them is bleeding. Monica alternates between periods of lucidity and periods of spatial and temporal confusion. The Emergency Room physicians informed Roberta of the clinical evolution but deferred a fuller discussion to the follow-up appointment scheduled for today with her referring oncologist. Neither chemotherapy nor radiotherapy is any longer indicated, owing to the bleeding brain lesion. The care plan involves referral of Monica to the palliative care team. The physician and the nurse meet Monica and Roberta at today’s follow-up visit.

## References

[1] Dohrenwend AM. Defining empathy to better teach, measure, and understand its impact. Acad Med. (2018) 93:1754–6. doi: 10.1097/ACM.0000000000002427, 30134271

[2] Rogers CR. A theory of therapy, personality, and interpersonal relationships, as developed in the client-centered framework. In: Koch S, ed. Psychology: A Study of a Science. 3. New York: McGraw-Hill (1959). 184–256.

[3] Halpern J. What is clinical empathy? J Gen Intern Med. (2003) 18:670–4. doi: 10.1046/j.1525-1497.2003.21017.x, 12911651 PMC1494899

[4] Hojat M Maio V Pohl CA Gonnella JS. Clinical empathy: definition, measurement, correlates, group differences, erosion, enhancement, and healthcare outcomes. Discov Health Syst. (2023) 2:8. doi: 10.1007/s44250-023-00020-2

[5] Weaver MF Jarvis MA Schnoll SH. Role of the primary care physician in problems of substance abuse. Arch Intern Med. (1999) 159:913–24. doi: 10.1001/archinte.159.9.913, 10326934

[6] Anzaldua A Halpern J. Can clinical empathy survive? Distress, burnout, and malignant duty in the age of Covid-19. Hastings Cent Rep. (2021) 51:22–7. doi: 10.1002/hast.1216, 33630324 PMC8013970

[7] Lamiani G Dordoni P Vegni E Barajon I. Caring for critically ill patients: clinicians’ empathy promotes job satisfaction and does not predict moral distress. Front Psychol. (2020) 10:2902. doi: 10.3389/fpsyg.2019.02902, 31969851 PMC6960200

[8] Wilkinson H Whittington R Perry L Eames C. Examining the relationship between burnout and empathy in healthcare professionals: a systematic review. Burn Res. (2017) 6:18–29. doi: 10.1016/j.burn.2017.06.003, 28868237 PMC5534210

[9] Zhang X Pang H f Duan Z. Educational efficacy of medical humanities in empathy of medical students and healthcare professionals: a systematic review and meta-analysis. BMC Med Educ. (2023) 23:925. doi: 10.1186/s12909-023-04932-8, 38057775 PMC10698992

[10] Bauchat JR Seropian M Jeffries PR. Communication and empathy in the patient-centered care model-why simulation-based training is not optional. Clin Simul Nurs. (2016) 12:356–9. doi: 10.1016/j.ecns.2016.04.003

[11] Neumann M Edelhäuser F Tauschel D Fischer MR Wirtz M Woopen C . Empathy decline and its reasons: a systematic review of studies with medical students and residents. Acad Med. (2011) 86:996–1009. doi: 10.1097/ACM.0b013e318221e615, 21670661

[12] Baile WF De Panfilis L Tanzi S Moroni M Walters R Biasco G. Using sociodrama and psychodrama to teach communication in end-of-life care. J Palliat Med. (2012) 15:1006–10. doi: 10.1089/jpm.2012.0030, 22799884

[13] Cleland JA Abe K Rethans JJ. The use of simulated patients in medical education: AMEE guide no. 31:42. Med Teach Informa Healthcare. (2009):477–86. doi: 10.1080/01421590903002821, 19811162

[14] Back A Arnold RM Baile WF Fryer-Edwards KA Stewart AC Barley GE . Efficacy of communication skills training for giving bad news and discussing transitions to palliative care. Arch Intern Med. (2007) 167:453–60. doi: 10.1001/archinte.167.5.453, 17353492

[15] Bays AM Engelberg RA Back AL Ford DW Downey L Shannon SE . Interprofessional communication skills training for serious illness: evaluation of a small-group, simulated patient intervention. J Palliat Med. (2014) 17:159–66. doi: 10.1089/jpm.2013.0318, 24180700 PMC4047839

[16] Bosméan L Chaffanjon P Bellier A. Impact of physician–patient relationship training on medical students’ interpersonal skills during simulated medical consultations: a cross-sectional study. BMC Med Educ. (2022) 22:117. doi: 10.1186/s12909-022-03171-7, 35193554 PMC8862366

[17] Meyer EC Brodsky D Hansen AR Lamiani G Sellers DE Browning DM. An interdisciplinary, family-focused approach to relational learning in neonatal intensive care. J Perinatol. (2011) 31:212–9. doi: 10.1038/jp.2010.109, 20706191

[18] Borghi L Meyer EC Vegni E Oteri R Almagioni P Lamiani G. Twelve years of the italian program to enhance relational and communication skills (Percs). Int J Environ Res Public Health. (2021) 18:1–14. doi: 10.3390/ijerph18020439, 33429873 PMC7826793

[19] Browning DM Salomon MZ. Relational learning in pediatric palliative care: transformative education and the culture of medicine. Child Adolesc Psychiatr Clin N Am. (2006) 15:795–815. doi: 10.1016/j.chc.2006.03.002, 16797450

[20] Hafferty FW Franks R. The hidden curriculum, ethics teaching, and the structure of medical education. Acad Med. (1994) 69:861–71. doi: 10.1097/00001888-199411000-00001, 7945681

[21] Haidet P Stein HF. The role of the student-teacher relationship in the formation of physicians: the hidden curriculum as process. J Gen Intern Med. (2006) 21:S16–20. doi: 10.1111/j.1525-1497.2006.00304.x, 16405704 PMC1484835

[22] Browning DM Meyer EC Truog RD Solomon MZ. Difficult conversations in health care: cultivating relational learning to address the hidden curriculum. Acad Med. (2007) 82:905–13. doi: 10.1097/ACM.0b013e31812f77b9, 17726405

[23] Epstein RM Hundert EM. Defining and assessing professional competence. JAMA. (2002) 287:226–35. doi: 10.1001/jama.287.2.226, 11779266

[24] Zoppi K Epstein RM. Is communication a skill? Communication behaviors and being in relation. Fam Med. (2002) 34:319–24. 12038712

[25] Kolb D. Experiential Learning: Experience as the Source of Learning and Development. Englewood Cliffs (NJ): Prentice-Hall (1984).

[26] Issenberg SB McGaghie WC Petrusa ER Lee Gordon D Scalese RJ. Features and uses of high-fidelity medical simulations that lead to effective learning: a BEME systematic review. Med Teach. (2005) 27:10–28. doi: 10.1080/01421590500046924, 16147767

[27] Fanning RM Gaba DM. The role of debriefing in simulation-based learning. Simul Healthc. (2007) 2:115–25. doi: 10.1097/SIH.0b013e3180315539, 19088616

[28] Mann K Gordon J MacLeod A. Reflection and reflective practice in health professions education: a systematic review. Adv Health Sci Educ Theory Pract. (2009) 14:595–621. doi: 10.1007/s10459-007-9090-2, 18034364

[29] Banerjee SC Manna R Coyle N Penn S Gallegos TE Zaider T . The implementation and evaluation of a communication skills training program for oncology nurses. Transl Behav Med. (2017) 7:615–23. doi: 10.1007/s13142-017-0473-5, 28211000 PMC5645276

[30] Ak M Cinar O Sutcigil L Congologlu ED Haciomeroglu B Canbaz H . Communication skills training for emergency nurses. Int J Med Sci. (2011) 8:397–401. doi: 10.7150/ijms.8.397, 21750643 PMC3133844

[31] Avlogiari E Maria Karagiannaki S Panteris E Konsta A Diakogiannis I. Improvement of medical students' empathy levels after an intensive experiential training on empathy skills. Psychiatry Clin Psychopharmacol. (2021) 31:392–400. doi: 10.5152/pcp.2021.21098, 38765648 PMC11079646

[32] Rasheed SP Younas A Sundus A. Self-awareness in nursing: a scoping review. J Clin Nurs. (2019) 28:762–74. doi: 10.1111/jocn.14708, 30362645

[33] Krasner MS Epstein RM Beckman H Suchman AL Chapman B Mooney CJ . Association of an educational program in mindful communication with burnout, empathy, and attitudes among primary care physicians. JAMA. (2009) 302:1284–93. doi: 10.1001/jama.2009.1384, 19773563

[34] Brown MEL MacLellan A Laughey W Omer U Himmi G LeBon T . Can stoic training develop medical student empathy and resilience? A mixed-methods study. BMC Med Educ. (2022) 22:340. doi: 10.1186/s12909-022-03391-x, 35505329 PMC9064267

[35] Geller G Grbic D Andolsek KM Caulfield M Roskovensky L. Article navigation journal article tolerance for ambiguity among medical students: patterns of change during medical school and their implications for professional development. Acad Med. (2021) 96:1036–42. doi: 10.1097/ACM.0000000000003820, 33149092

[36] Lamiani G Meyer EC Leone D Vegni E Browning DM Rider EA . Cross-cultural adaptation of an innovative approach to learning about difficult conversations in healthcare. Med Teach. (2011) 33:e57–64. doi: 10.3109/0142159X.2011.534207, 21275534

[37] Hojat M Mangione S Nasca TJ Rattner S Erdmann JB Gonnella JS . An empirical study of decline in empathy in medical school. Med Educ. (2004) 38:934–41. doi: 10.1111/j.1365-2929.2004.01911.x, 15327674

[38] Hojat M DeSantis J Shannon SC Speicher MR Bragan L Calabrese LH. Empathy as related to gender, age, race and ethnicity, academic background and career interest: a nationwide study of osteopathic medical students in the United States. Med Educ. (2020) 54:571–81. doi: 10.1111/medu.14138, 32083747 PMC7317910

[39] Hojat M Gonnella JS Mangione S Nasca TJ Veloski JJ Erdmann JB . Empathy in medical students as related to academic performance, clinical competence and gender. Med Educ. (2002) 36:522–7. doi: 10.1046/j.1365-2923.2002.01234.x, 12047665

[40] Hojat M Gonnella JS Nasca TJ Mangione S Vergare M Magee M. Physician empathy: definition, components, measurement, and relationship to gender and specialty. Am J Psychiatry. (2002) 159:1563–9. doi: 10.1176/appi.ajp.159.9.1563, 12202278

[41] Stegmann K Pilz F Siebeck M Fischer F. Vicarious learning during simulations: is it more effective than hands-on training? Med Educ. (2012) 46:1001–8. doi: 10.1111/j.1365-2923.2012.04344.x, 22989134

[42] O’Regan S Molloy E Watterson L Nestel D. Observer roles that optimise learning in healthcare simulation education: a systematic review. Adv Simul (Lond). (2016) 1:4. doi: 10.1186/s41077-015-0004-8, 29449973 PMC5796608

[43] Lamiani G. Meyer E. C. Browning D. M. Moja E. A. Tra scienza e sofferenza: le conversazioni difficili in sanità [between science and suffering: difficult conversations in healthcare]. Recenti Prog Med (2009) 100:239–246. doi: 10.1701/427.5080 19772214

[44] Hojat M. Empathy in Health Professions Education and Patient Care. Cham: Springer (2016).

[45] Hojat M Mangione S Nasca TJ Cohen MJM Gonnella JS Erdmann JB . The Jefferson scale of physician empathy: development and preliminary psychometric data. Educ Psychol Meas. (2001) 61:349–65. doi: 10.1177/00131640121971158

[46] Di Lillo M Cicchetti A Lo Scalzo A Taroni F Hojat M. The Jefferson scale of physician empathy: preliminary psychometrics and group comparisons in Italian physicians. Acad Med. (2009) 84:1198–202. doi: 10.1097/ACM.0b013e3181b17b3f, 19707057

[47] Byrne M Campos C Daly S Lok B Miles A. The current state of empathy, compassion and person-centred communication training in healthcare: an umbrella review. Patient Educ Couns. (2024) 119:108063. doi: 10.1016/j.pec.2023.108063, 38008647

[48] Bansal A Greenley S Mitchell C Park S Shearn K Reeve J. Optimising planned medical education strategies to develop learners' person-centredness: a realist review. Med Educ. (2022) 56:489–503. doi: 10.1111/medu.14707, 34842290 PMC9306905

[49] Levett-Jones T Cant R Lapkin S. A systematic review of the effectiveness of empathy education for undergraduate nursing students. Nurse Educ Today. (2019) 75:80–94. doi: 10.1016/j.nedt.2019.01.006, 30739841

[50] Hojat M Gonnella JS Nasca TJ Mangione S Veloksi JJ Magee M. The Jefferson scale of physician empathy: further psychometric data and differences by gender and specialty at item level. Acad Med. (2002) 77:S58–60. doi: 10.1097/00001888-200210001-00019, 12377706

[51] Miguez-Torres N Martínez-Rodríguez A Martínez-Olcina M Miralles-Amorós L Reche-García C. Relationship between emotional intelligence, sleep quality and body mass index in emergency nurses. Healthcare (Basel). (2021) 9:607. doi: 10.3390/healthcare9050607, 34070223 PMC8158709

[52] Bru-Luna LM Martí-Vilar M Merino-Soto C Cervera-Santiago JL. Emotional intelligence measures: a systematic review. Healthcare (Basel). (2021) 9:1696. doi: 10.3390/healthcare9121696, 34946422 PMC8701889

[53] Fallowfield L Lipkin M Hall A. Teaching senior oncologists communication skills: results from phase I of a comprehensive longitudinal program in the United Kingdom. J Clin Oncol. (1998) 16:1961–8. doi: 10.1200/JCO.1998.16.5.1961, 9586916

[54] Howick J Dudko M Feng SN Ahmed AA Alluri N Nockels K . Why might medical student empathy change throughout medical school? A systematic review and thematic synthesis of qualitative studies. BMC Med Educ. (2023) 23:270. doi: 10.1186/s12909-023-04165-9, 37088814 PMC10124056

[55] Moudatsou M Stavropoulou A Philalithis A Koukouli S. The role of empathy in health and social care professionals. Healthcare (Switzerland). (2020) 8:26. doi: 10.3390/healthcare8010026, 32019104 PMC7151200

[56] Stephens E William L Lim LL Allen J Zappa B Newnham E . Complex conversations in a healthcare setting: experiences from an interprofessional workshop on clinician-patient communication skills. BMC Med Educ. (2021) 21:343. doi: 10.1186/s12909-021-02785-7, 34126985 PMC8204413

[57] Brighton LJ Selman LE Gough N Nadicksbernd JJ Bristowe K Millington-Sanders C . Difficult conversations’: evaluation of multiprofessional training. BMJ Support Palliat Care. (2018) 8:45–8. doi: 10.1136/bmjspcare-2017-001447, 29118100 PMC5867425

[58] Nembhard IM David G Ezzeddine I Betts D Radin J. A systematic review of research on empathy in health care. Health Serv Res. (2023) 58:250–63. doi: 10.1111/1475-6773.14016, 35765156 PMC10012244

[59] Rathert C Wyrwich MD Boren SA. Patient-centered care and outcomes: a systematic review of the literature. Med Care Res Rev. (2013) 70:351–79. doi: 10.1177/1077558712465774, 23169897

[60] Robinson JH Callister LC Berry JA Dearing KA. Patient-centered care and adherence: definitions and applications to improve outcomes. J Am Acad Nurse Pract. (2008) 20:600–7. doi: 10.1111/j.1745-7599.2008.00360.x, 19120591

